# Unique copper and reduced graphene oxide nanocomposite toward the efficient electrochemical reduction of carbon dioxide

**DOI:** 10.1038/s41598-017-03601-3

**Published:** 2017-06-09

**Authors:** M. Nur Hossain, Jiali Wen, Aicheng Chen

**Affiliations:** Department of Chemistry, Lakehead University, 955 Oliver Road, Thunder Bay, ON P7B 5E1 Canada

## Abstract

The electrochemical reduction of CO_2_ to useful chemicals and fuels has garnered a keen and broad interest. Herein, we report a unique nanocomposite consisting of Cu nanoparticles (NPs) and reduced graphene oxide (rGO) supported on a Cu substrate with a high catalytic activity for CO_2_ reduction. The nanocomposite was optimized in terms of the composition of Cu NPs and rGO as well as the overall amount. A gas chromatograph was employed to analyze the gaseous products, whereas a chemical oxygen demand (COD) method was proposed and utilized to quantify the overall liquid products. The optimized nanocomposite could effectively reduce CO_2_ to CO, HCOOH and CH_4_ with a Faradaic efficiency (FE) of 76.6% at −0.4 V (vs. RHE) in a CO_2_ saturated NaHCO_3_ solution. The remarkable catalytic activity, high FE, and excellent stability make this Cu-rGO nanocomposite promising for the electrochemical reduction of CO_2_ to value-added products to address the pressing environmental and energy challenges.

## Introduction

Increasing levels of CO_2_ in the atmosphere have created a highly concerning situation that continues to elevate global average temperatures. There is a growing frequency of reports related to the impacts of global climate change due to increasing greenhouse gas emissions via the continuous combustion of fossil fuels. One of the most notorious of the greenhouse gases is CO_2_, which is released by both natural and anthropogenic processes. There is a great interest in capture and sequestration of CO_2_ emissions prior to their release into the ambient atmosphere, or the conversion of this gas to useful products such as fuels^1–5^. Over the last few decades, various electrocatalysts have been explored for the electrochemical reduction of CO_2_ to valuable fuels^6–10^, and a wide range of gas and liquid products may be formed. Therefore, it is essential to accurately determine both the produced gases and the liquid fuels in order to precisely assess the FE. On one hand, the primary gas products include CO, methane (CH_4_), and ethane, which may be determined using GC and gas chromatography mass spectrometry (GC-MS)^11, 12^. On the other hand, potential liquid products include formate, acetate, aldehyde, alcohols, and so on, which strongly depend on the electrocatalysts employed and the applied electrode potentials. Although high performance liquid chromatography (HPLC), ion chromatography (IC) and nuclear magnetic resonance (NMR) have been employed to analyze the liquid products^11, 13–15^, it remains quite challenging and time-consuming to quantify the various liquid products in order to determine the overall FE and assess the activity of the catalysts. A chemical oxygen demand (COD) method is commonly used in environmental analysis, and is based upon the complete oxidation of all organic species to CO_2_, which is exactly the reverse of the CO_2_ reduction process^16, 17^. In this study, for the first time we propose and employ the COD analysis to determine the overall FE associated with the conversion of CO_2_ to liquid chemicals and fuels.

Graphene nanosheets have been widely doped and/or modified for catalytic and energy conversion applications^18–22^. The unique electronic and physical properties of graphene may augment the reduction kinetics of CO_2_, and enhance the reaction kinetics of noble metal nanoparticles^23, 24^. Copper is considered to be one of the eminent catalysts for the electrochemical reduction of CO_2_ to low-carbon fuels for high-density renewable energy storage^7, 8, 11, 25^. It has been reported that CO, CH_4_, C2 hydrocarbon, alcohols, formate, and acetate could be formed when Cu was used as an electrode in an aqueous solution^11, 12, 25–27^. Several studies have been reported wherein the selectivity of Cu catalysts for the reduction of CO_2_ was specifically dependent on its crystal facets^7, 28, 29^. The surface structures of Cu electrodes, in conjunction with the applied electrode potential, are of intense interest for product selectivity^30–33^. Density Function Theory studies have indicated that defective graphene-supported Cu nanoparticles may modify the structural and electronic properties of copper, toward enhancing the electrochemical reduction of CO_2_ to fuels (e.g., CH_4_, CO, and HCOOH)^23, 24, 34–38^. However, despite the high catalytic activity of such Cu catalysts, they still suffer from low stability and large reaction overpotentials. Herein we report on the high-performance CO_2_ reduction that is enabled by a unique nanocomposite of Cu NPs and rGO supported on a Cu substrate with high FE and stability for the efficient conversion of CO_2_ to valuable fuels, including CO, CH_4_, and formate.

## Results

### Syntheses and characterization of Cu-rGO nanocomposites

**Figure 1:**
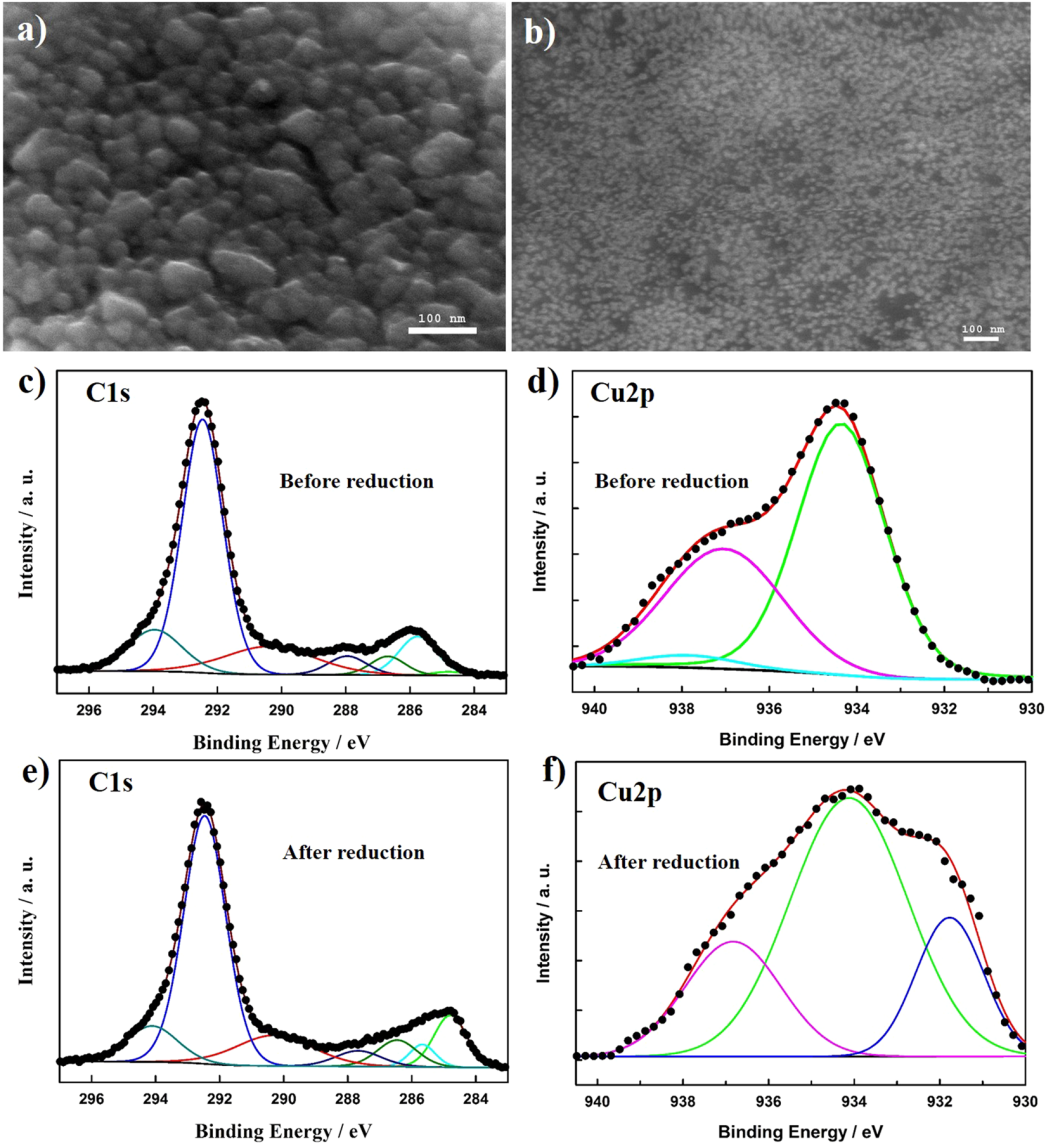
SEM images of the formed Cu NPs (**a**) and the Cu-rGO nanocomposite (**b**) on a Cu substrate. High-resolution XPS spectra of the C1s region (**c** and **e**) and the Cu2p region (**d** and **f**) of the CuSO_4_-GO thin film before the electrochemical treatment and the formed Cu-rGO nanocomposite.

The Cu-rGO nanocomposite was formed directly on a Cu substrate using a facile electrochemical reduction method. A mixture of GO and Cu^2+^ precursors was cast on an etched Cu substrate; and the simultaneous formation of Cu-rGO nanocomposite was achieved via cyclic voltammetry (CV), which was carried out in 0.1 M Na_2_SO_4_ in the potential range from 0.62 to −0.58 V vs. RHE for five cycles. The composition and thickness of the formed Cu-rGO nanocomposite were also optimized, with the experimental details described in the Methods. Figure 1a and b display the scanning electron microscope (SEM) images of the formed Cu NPs in the absence of GO, and the Cu-rGO nanocomposite, respectively. It is evident that large grain-sized Cu particles were formed in the absence of GO. In contrast, Cu NPs with an average diameter of ~10 nm were distributed homogeneously on the rGO. Energy dispersive X-ray spectra (Supplementary Fig. S1) exhibited a strong Cu peak for the Cu NPs (Curve i) and an additional strong C peak for the Cu-rGO nanocomposite electrode (Curve ii). X-ray photoelectron spectroscopic (XPS) measurements were further carried out for mixture of the GO and Cu precursor mixture as well as for the formed Cu-rGO nanocomposite electrode. Figure 1c and e display the high-resolution C1s XPS spectra prior to and following the electrochemical reduction, respectively. A series of fitting peaks were observed at 284.80, 285.76, 286.69, 287.95, and 290.39 eV, corresponding to sp^2^ C, C-OH, C-O, C=O, and HO-C=O bonds, respectively, as observed in GO^21, 22^. The peaks centred at 292.46 and 293.96 eV are due to the C-F_3_ and C-F_2_ groups of Nafion, which was used as the binding material of the nanocomposite to the substrate. As seen in Table S1, following the electrochemical reduction, the peaks of oxygen-containing groups decreased; and the proportion of C=C group increased enormously, revealing that the electrochemical treatment had a significant effect on the diminution of oxygen-containing functional groups. In the case of Cu, prior to the electrochemical treatment, three Cu2p peaks were observed in Fig. 1d, which might be attributed to the physicochemical interactions of Cu(II) species with the different functional groups of GO^15, 39–41^. Subsequent to the electrochemical treatment (Fig. 1f), a strong Cu(0) peak appeared at 934.12 eV and a small Cu(I) peak was observed at 931.78 eV. The associated Cu2p peak position, assignment, and atomic percentage before and after the electrochemical treatment are listed in Table S2. All the aforementioned results show that the GO and Cu^2+^ precursor can be effectively reduced to form the Cu-rGO nanocomposite.

**Figure 2:**
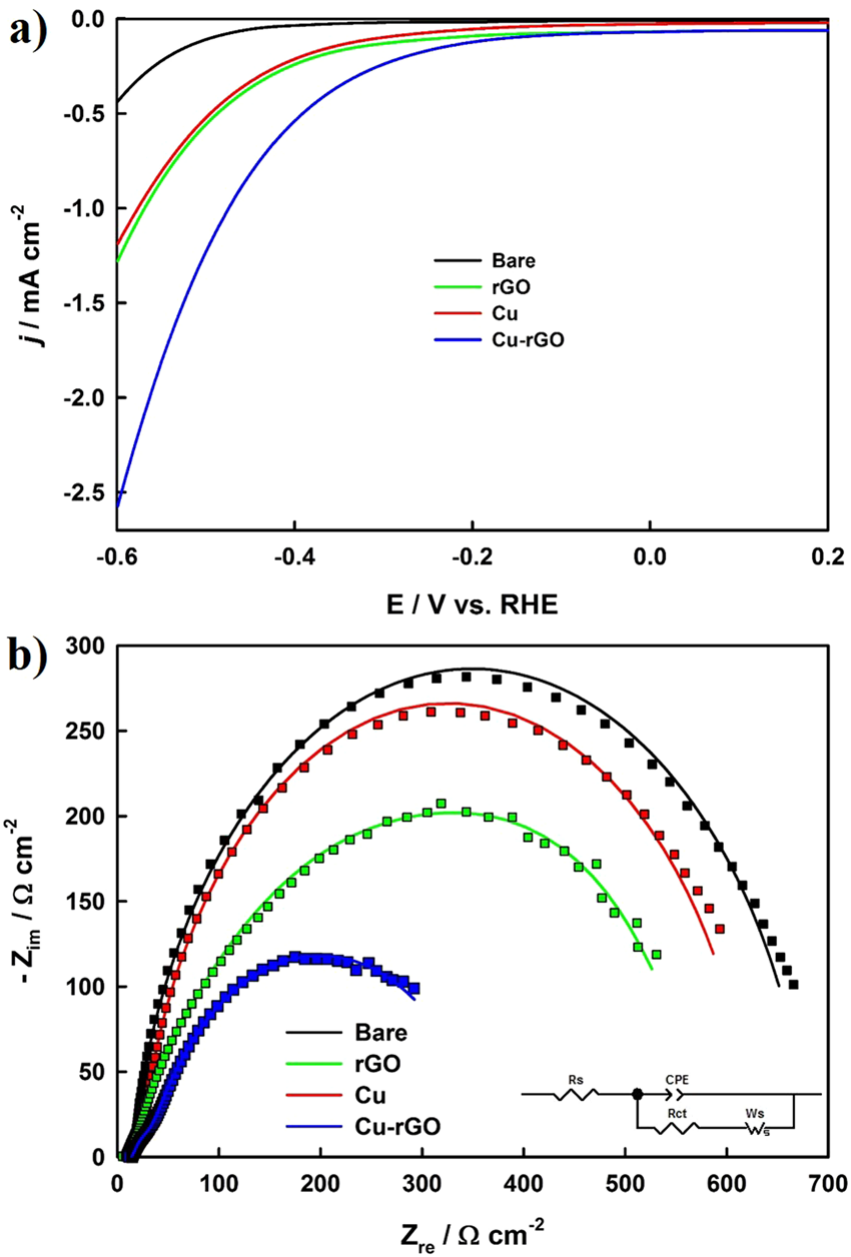
(**a**) LSV curves of the bare Cu substrate, rGO, Cu NPs and Cu-rGO nanocomposite electrodes; and (**b**) the corresponding Nyquist plots measured at the potential of −0.4 V in a CO_2_-saturated 0.1 M NaHCO_3_ solution. Inset: the equivalent electric circuit used for fitting the EIS data, where Rs = solution resistance; CPE = constant phase element; Rct = charge-transfer resistance; Ws = Warburg impedance (short).

The electrocatalytic activity of the formed Cu-rGO nanocomposite was initially studied using linear sweep voltammetry (LSV) and chronoamperometry (CA) in the presence of CO_2_ in 0.1 M NaHCO_3_ (pH 6.65). Figure 2a compares the LSV curves of the bare Cu substrate, Cu NPs, rGO, and the Cu-rGO nanocomposite recorded at 20 mV s^−1^. The Cu-rGO nanocomposite exhibited a much higher current density and earlier onset potential in contrast to the bare Cu substrate, Cu NPs, and rGO. The CA curves of these electrodes were measured at −0.4 V and compared in Supplementary Fig. S2, showing that the steady-state current was increased in the following order: bare Cu < rGO ≈ Cu NPs < Cu-rGO. It is noteworthy that the current density of the Cu-rGO nanocomposite was much higher, and the onset potential was much lower in comparison with other copper-based catalysts for the electroreduction of CO_2_ that have been recently reported in the literature^36–38, 42–44^.

Electrochemical impedance spectroscopy (EIS) was employed to determine the charge-transfer resistance. Nyquist plots (Fig. 2b) of the bare Cu, Cu NPs, rGO, and Cu-rGO nanocomposite electrodes were recorded in CO_2_-saturated 0.1 M NaHCO_3_ aqueous solutions at −0.4 V. All of the impedance curves exhibited a semi-circle in the low-frequency region, which may correspond to the charge transfer resistance of the CO_2_ reduction^45^. The equivalent electrical circuit displayed in the inset was employed to fit the experimental data using the Z-view software, and the corresponding fitted results were summarized along with the percentage of errors in Supplementary Table S3. All of the solution resistances (Rs) were small, and the low error percentages indicated that the employed equivalent circuit fitted the impedance data well. All of the CPE-P values were >0.8, which signified that the constant phase element (CPE) behaviours were capacitor-like. The Cu-rGO nanocomposite exhibited the highest CPE-T value (1817.60 μF cm^−2^), which was over four-fold larger than that of the Cu NPs (427.38 μF cm^−2^) and over two-fold greater than that of the rGO (781.40 μF cm^−2^). Moreover, the Cu-rGO nanocomposite exhibited much lower charge-transfer resistance Rct (355.40 Ω cm^−2^), which was almost half of the Cu NPs (612.90 Ω cm^−2^). A short Warburg impedance (Ws) associated with Rct was included in the equivalent circuit in order to effectively fit the impedance spectra, indicating that the diffusion resistance also played an important role during the electrochemical reduction of CO_2_. All of the EIS results further demonstrated that the Cu-rGO nanocomposite exhibited much higher catalytic activity toward the electrochemical reduction of CO_2_ in comparison to the Cu NPs and rGO.

**Figure 3:**
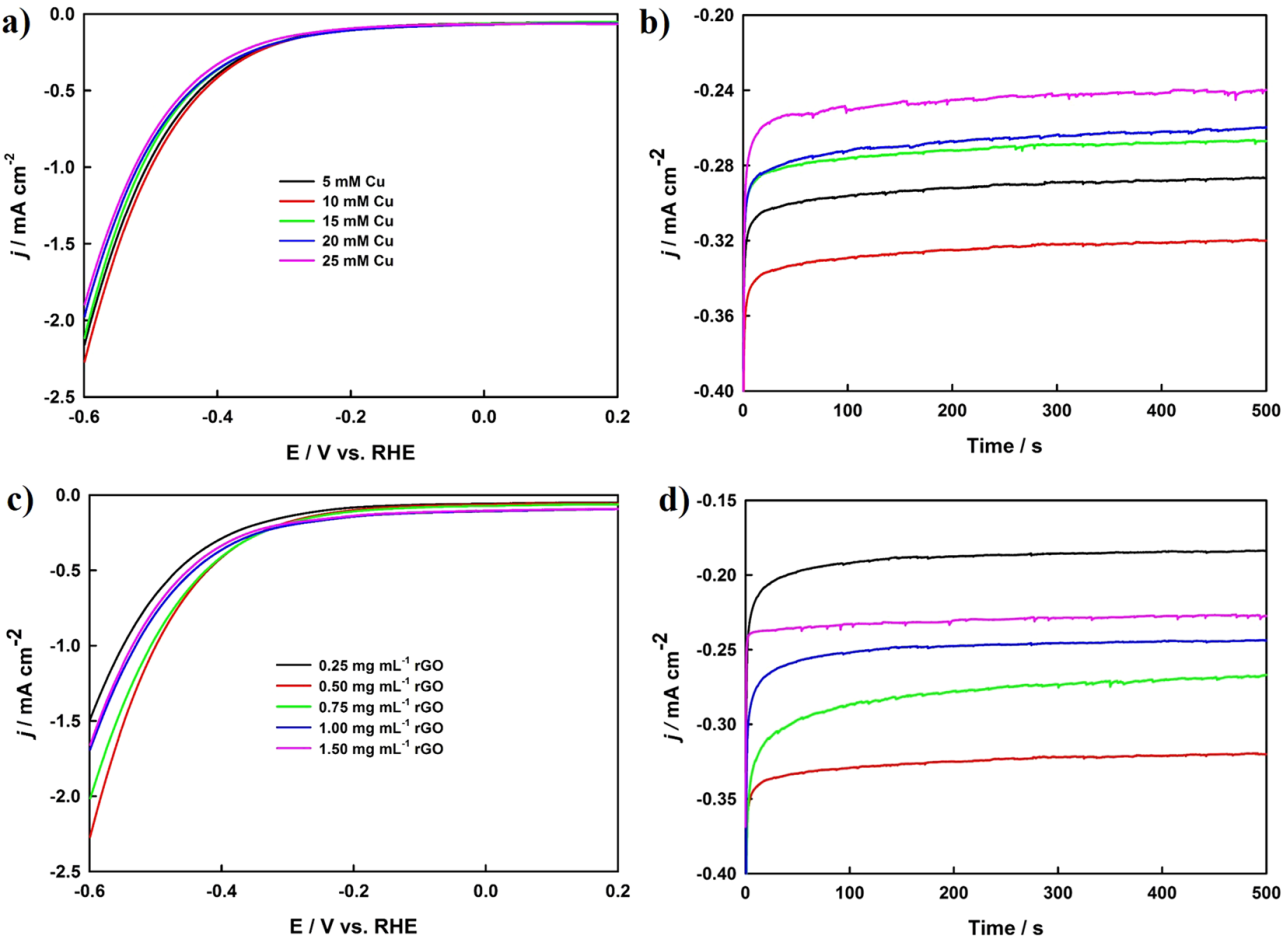
LSV curves (**a**) and CA plots (**b**) of the Cu-rGO nanocomposite electrodes prepared with a constant GO concentration (0.5 mg mL^−1^) while the concentration of the Cu precursor was changed from 5 to 25 mM as listed in Fig. 3a. LSV curves (**c**) and CA plots (**d**) for the optimization of the GO concentration while the concentration of the Cu precursor was maintained at 10 mM.

**Figure 4:**
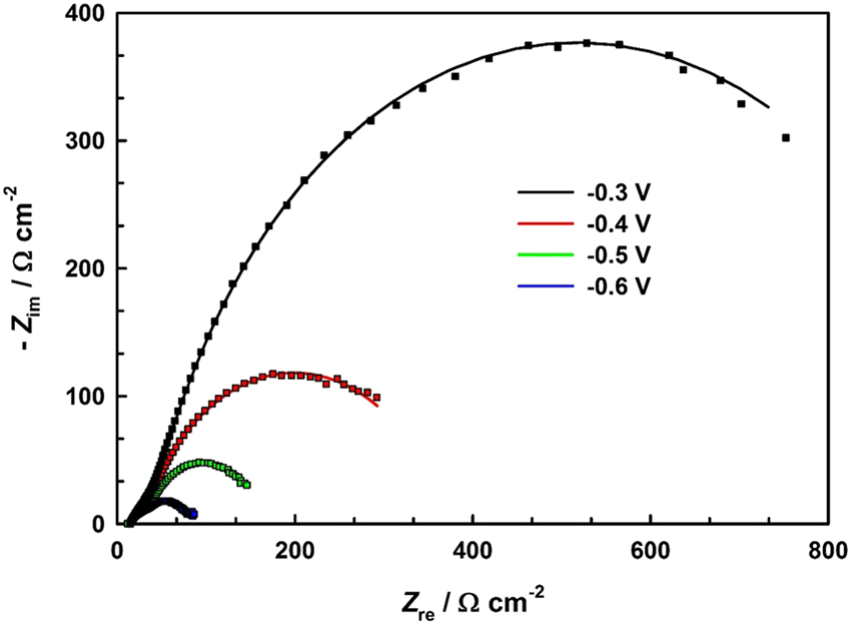
Nyquist plots of the optimized Cu-rGO nanocomposite electrode recorded at −0.3, −0.4, −0.5, and −0.6 V in a CO_2_ saturated 0.1 M NaHCO_3_ solution. The symbols denote the experimental data and the solid lines correspond to the fitted results using the equivalent electrical circuit (inset). The amplitude of the modulation potential was 10 mV and the frequency was altered from 100 kHz to 10 mHz.

In order to optimize the composition and quantity of the nanocomposite, different Cu-rGO nanocomposites were prepared and studied. Figure 3 presents the LSV and CA curves of the prepared Cu-rGO nanocomposites, where the concentration of the Cu precursor was altered from 5 to 25 mM, and the GO concentration was varied from 0.25 to 1.5 mg mL^−1^. As shown in both LSV (Fig. 3a) and CA (Fig. 3b) plots, the highest current density was achieved with the 10.0 mM Cu precursor. In the case of GO, the highest activity was observed when 0.5 mg mL^−1^ GO was used, as seen in Fig. 3c and d. To study the effects of the thickness of the nanocomposite, similar experiments were conducted with the optimized composite mixture of the 10 mM Cu precursor and 0.5 mg mL^−1^ GO, where the volume was changed from 25 to 150 μL. The highest current density was attained with 75 μL of the composite mixture, as shown in both the LSV and CA curves (Supplementary Information Fig. S3a and S3b). EIS was further carried out to investigate the charge-transfer resistance of the optimized Cu-rGO nanocomposite (75 μL of 10 mM Cu and 0.5 mg mL^−1^ GO mixture) at four different applied electrode potentials (Fig. 4). The EIS curves were effectively fitted with the electrical circuit (inset of Fig. 4) with the results listed in Table S4 (Supplementary Information), revealing that the charge-transfer resistance was significantly decreased from 976.0 to 70.26 Ω cm^−2^, with the increase of the cathodic potential from −0.3 to −0.6 V.

### Bulk electrolysis of CO_2_

**Figure 5:**
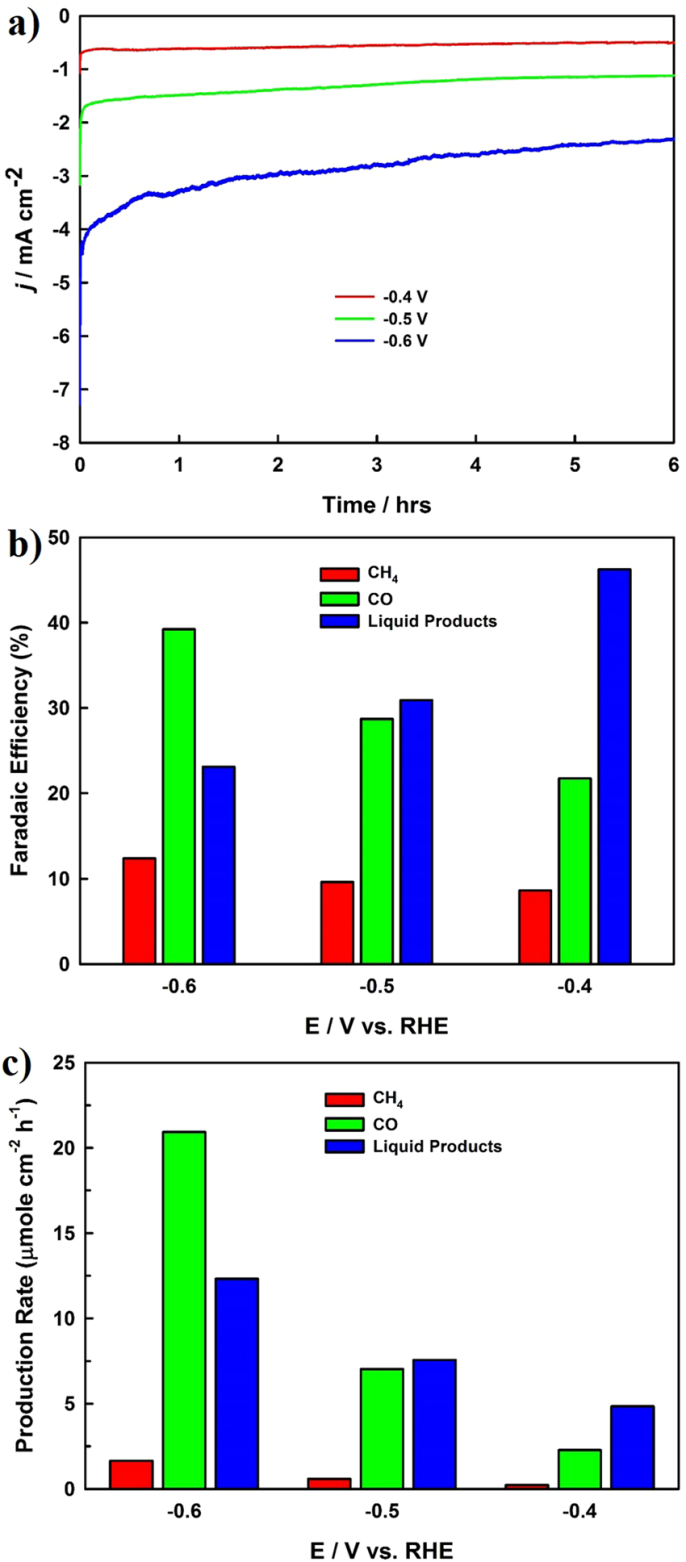
(**a**) CA curves of the optimized Cu-rGO nanocomposite recorded at the selected potentials of −0.4, −0.5, and −0.6 V for product analysis. (**b**) The corresponding Faradaic efficiency of the formed products at the different applied potentials over the six hours. (**c**) The rates of product formation during the electrochemical reduction of CO_2_ at the applied potentials on the Cu-rGO nanocomposite electrode.

In an attempt to achieve the bulk electrolysis of CO_2_, we selected three potentials (−0.4, −0.5, and −0.6 V) for six hours of electrolysis using the optimized Cu-rGO nanocomposite in a CO_2_-saturated 0.1 M NaHCO_3_ electrolyte (pH 6.65). Figure 5a shows that the current density increased with the elevation of the cathode potentials during the bulk electrolysis of CO_2_, which was indicative of accelerated CO_2_ reduction reaction rates at more negative potentials. The formation of a large quantity of product was also observed at more negative potentials, revealing that the Cu-rGO nanocomposite facilitated the charge-transfer for the CO_2_ reduction, while increasing the cathodic potential. Our GC analysis showed that CO and CH_4_ were the primary gas products generated from the electrochemical reduction of CO_2_. To confirm that whether rGO served as the potential carbon source, we have conducted two control experiments: (i) running the CA experiment at −0.5 V vs RHE for six hours in an Ar-saturated 0.1 M NaHCO_3_ solution using the rGO electrode; and (ii) performing the same CA test in an Ar-saturated 0.1 M Na_2_SO_4_ solution using the Cu-rGO nanocomposite. Only hydrogen was detected in the GC analysis for both cases, confirming that the CO and CH_4_ products were formed from the electrochemical reduction of CO_2_ using the Cu-rGO nanocomposite as the electrocatalyst. The FE for the formation of CO and CH_4_ at the different applied electrode potentials were calculated and plotted in Fig. 5b, showing that more CO was generated than CH_4_ and that the FE for the formation of the gas products was increased with the raising of the cathodic potential from −0.4 to −0.6 V.

As mentioned in the introduction, the COD analysis is just the opposite of the CO_2_ reduction; and this reverse conversion allows us to rapidly determine the total amount of electrons that are used in the electrochemical reduction of CO_2_ to produce the various organic liquid fuels as they will be completely oxidized via the following general equation during the COD analysis^16, 17^:

1 $${{\rm{C}}}_{{\rm{a}}}{{\rm{H}}}_{{\rm{b}}}{{\rm{O}}}_{{\rm{c}}}+({\rm{a}}+{\rm{b}}/4-{\rm{c}}/2)\,{{\rm{O}}}_{2}\to {\rm{a}}\,{{\rm{CO}}}_{2}+{\rm{b}}/2{{\rm{H}}}_{2}{\rm{O}}$$

where a, b, and c represent the stoichiometric ratio of carbon, hydrogen, and oxygen in the formed organic compounds, respectively. Since each O_2_ molecule corresponds to a four-electron transfer,

2 $${{\rm{O}}}_{2}+4{{\rm{H}}}^{+}+4{{\rm{e}}}^{-}\to 2{{\rm{H}}}_{2}{\rm{O}}$$

we may use the following equation to calculate the total charge (Q_COD_), which was consumed for the formation of the liquid fuels during the electrochemical reduction of CO_2_:

3 $${{\rm{Q}}}_{{\rm{C}}{\rm{O}}{\rm{D}}}={\rm{C}}{\rm{O}}{\rm{D}}[{\rm{m}}{\rm{g}}\,{{\rm{L}}}^{-1}\,{{\rm{O}}}_{2}]\times (4FV/32000)$$

where *F* is the Faraday constant and *V* is the volume of the solution. Thus, the Faradaic efficiency for the formation of the liquid products may be calculated as follows:

4 $${{\rm{FE}}}_{{\rm{COD}}} \% ={{\rm{Q}}}_{{\rm{COD}}}/{\rm{Q}}\times 100$$

where *Q* is the overall charge passed during the electrochemical reduction of CO_2_. The FE_COD_ at the different potentials was calculated and plotted in Fig. 5b, showing that the FE in the formation of liquid chemicals/fuels was decreased when the cathodic potential was raised from −0.4 to −0.6 V. Our further HPLC analysis confirmed that the primary liquid product was formate with a trace amount of acetate. For comparison, the CA experiment was also performed at −0.5 V in an Ar-saturated 0.1 M Na_2_SO_4_ solution for six hours using the Cu-rGO nanocomposite. The subsequent COD analysis was carried out and no COD change of the solution was observed prior to and after the CA test, further confirming that the increase of the COD value in the electrolysis experiment performed in the CO_2_-saturated 0.1 M NaHCO_3_ solution was resulted from the electrochemical reduction of CO_2_. The total FE of the optimized Cu-rGO nanocomposite for the reduction of CO_2_ included two parts (the formation of the gas and liquid products); and it was calculated to be 76.6%, 69.2%, and 74.7% at the applied electrode potentials of −0.4, −0.5, and −0.6 V vs. RHE, respectively, and the rest ~25% might be attributed to the hydrogen evolution. The rates of the formation of the products at the different applied electrode potentials are presented in Fig. 5c, revealing that all the production rates were increased with the increase of the cathodic potential from −0.4 to −0.6 V and that a significant increase of the CO formation was observed in comparison of the formation of the other gas and liquid products. Various Cu-based materials have been explored for the electrochemical reduction of CO_2_, and some of the promising results recently reported in the literature are compared in Table 1, showing that the unique Cu-rGO nanocomposite developed in the present study exhibited excellent FE for the efficient electrocatalytic reduction of CO_2_ at a relative low overpotential.

**Table 1:** Comparison of some promising Cu-based catalysts for the electrochemical reduction of CO_2_ recently reported in the literature.

Electrocatalyst	Applied potential	Products (FE%)	Total FE%	Ref.
Oxide derived Cu	−0.40 V vs. RHE	CO (38.5); HCOO^−^ (10.8)	49.3	39
Cu nanowire	−0.795 V vs. RHE	CO (2.4); C_2_H_4_ (7.2); C_2_H_6_ (8.3); HCOO^−^ (9.6); CH_3_CH_2_OH (10.8)	38.64	46
Carbon nanotube/copper sheets (CNT/Cu)	−2.8 V vs. Ag/AgCl	CO (1.6); CH_4_ (6.8)	7.6	43
	−5.0 V vs. Ag/AgCl	CO (5.1); CH_4_ (15.5); C_2_H_4_ (1.1)	21.7	
Copper nanoparticles supported on carbon black (40 wt% Cu/VC)	−1.2 V vs. Ag/AgCl	CO (~15.0)	15.0	44
20% Cu/CNT	−1.7 V vs. SCE	CH_3_OH (38.4)	38.4	47
Carbon nanospike electrode with electronucleated Cu nanoparticles (Cu/CNS)	−0.7 V vs. RHE	CO (23.0); CH_4_ (12.0)	25.0	36
Graphene confined Sn quantum sheets	−1.2 V vs. SCE	HCOO^−^ (30.0)	30.0	48
Cu NPs loaded on glassy carbon (Cu NP/GC)	−1.3 V vs. RHE	CO (5.5); CH_4_ (40.1); C_2_H_4_ (2.3); HCOOH (1.9); CH_3_COOH (0.7)	50.5	35
Oxide derived Cu foam	−1.0 V vs. RHE	CO (~5.0); HCOO^−^ (~5.0); C_2_H_4_ (~20.0); C_2_H_6_ (~25.0)	55.0	49
Copper nanoparticles supported on glassy carbon (n-Cu/C)	−0.95 vs. RHE	CH_4_ (~15.0)	15.0	25
Cu mesocrystals	−0.99 V vs. RHE	CO (~2.0); CH_4_ (~3.0); C_2_H_4_ (~27.0)	32.0	50
Cu nanoflower	−1.6 V vs. RHE	HCOOH (~50.0); CH_4_ (~5.0); C_2_H_4_ (~10.0)	65.0	28
**Cu-rGO**	**−0.4 V vs. RHE (−1.0 V vs. Ag/AgCl)**	**CO (21.7); CH**_**4**_ **(8.6); Liquid products (46.2)**	**76.6**	**Present work**

**Figure 6:**
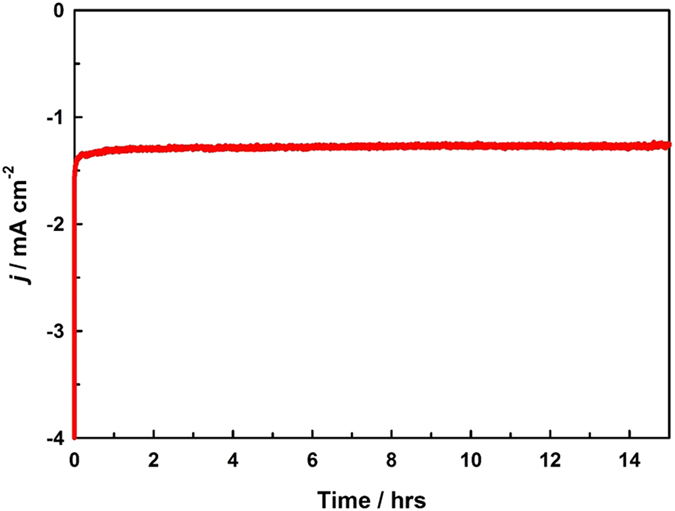
The stability test of the optimized nanocomposite electrode carried at −0.5 V in a CO_2_-saturated 0.1 M NaHCO_3_ solution under continuous CO_2_ purging.

Finally, the stability of the optimized Cu-rGO nanocomposite was tested at −0.5 V in one liter of CO_2_-saturated 0.1 M NaHCO_3_ over 15 hours, via the chronoamperometric method (Fig. 6), where CO_2_ was continuously purged into the solution during the course of the test. Impressively, the current density was almost identical during the entire CO_2_ reduction electrolyses, which demonstrated the high stability of the Cu-rGO nanocomposite electrode. The superior stability was further confirmed by inductively coupled plasma atomic emission spectroscopic (ICP-AES) analysis, where no Cu was detected in the electrolyte following the electrolysis.

## Discussion

A unique Cu-rGO nanocomposite has been developed in the present study as a high-performance electrocatalyst for the efficacious reduction of CO_2_ in an aqueous solution, with a high current density and a low cathodic potential. The superior electrocatalytic activity and stability of the Cu-rGO nanocomposite achieved in our study can be attributed to the uniformly distributed small Cu nanoparticles on the rGO and the synergistic coupling effect of the formed nanocomposite. The electron transfer between the rGO and Cu nanoparticles may increase localized electron concentrations, resulting in significant enhancement of the catalytic activities of the nanocomposite for the electrochemical reduction of CO_2_. The GC measurements indicated that CO and CH_4_ were the primary gas products, while our HPLC analysis revealed that HCOO^−^ was the dominant liquid product. In addition, we have employed COD analysis to quantify the overall liquid products, which provides a facile, rapid, and accurate method for the determination of the total FE for the conversion of CO_2_ to liquid products. The COD analysis may become a universal approach for quantification of the overall liquid products generated in other CO_2_ conversion processes, for instance, photochemical and photoelectrochemical reduction of CO_2_. The easy fabrication, cost-effectiveness, high intrinsic activity, superior stability, and excellent FE make the Cu-rGO nanocomposite developed in this study a very promising catalyst for the efficient electrochemical conversion of CO_2_ to valuable fuels. This might enable a new strategy for the restoration of the carbon balance, while contributing to the resolution of the climate change.

## Methods

### Materials

Graphene oxide, CuSO_4_.5H_2_O (99.999%), NaHCO_3_ (≥99.0%), Na_2_SO_4_ (≥99.5%) and a 10 wt.% Nafion solution were obtained from Sigma Aldrich. Copper foil (99.9985%, 0.5 mm thick) was purchased from Alfa Aesar; copper wire (99.9%, 1.0 mm diameter) was purchased from Sigma Aldrich; and carbon dioxide (99.9%) was purchased from Praxair. All electrochemical experiments were conducted in a 0.1 M NaHCO_3_ electrolyte solution under CO_2_ saturation. Double distilled water treated by a Nanopure Diamond water purification system (18 MΩ cm) was used in the preparation of all the solutions. All chemicals were used directly without further treatment.

### Synthesis of Cu-rGO nanocomposites

To optimize the concentration of Cu precursor, a 50 μL mixed solution of Nafion (0.5%), GO (0.5 mg mL^−1^) and CuSO_4_.5H_2_O (5.0, 10.0, 15.0, 20.0, and 25.0 mM) was cast on a 1.0 cm^2^ Cu foil, which was etched for 30 s in 35% HNO_3_, washed with deionized water, and dried. To optimize the concentration of GO, a 50 μL suspension of Nafion (0.5%), CuSO_4_.5H_2_O (10 mM), and GO (0.25, 0.5, 0.75, 1.0 and 1.50 mg mL^−1^) was cast onto the etched 1.0 cm^2^ Cu foil. Similarly, to optimize the Cu-rGO thickness, altered volumes of the suspension solution (25, 50, 75, 100 and 150 μL) containing 10.0 mM CuSO_4_.5H_2_O, 0.5% Nafion, and a 0.5 mg mL^−1^ GO were cast on the etched 1.0 cm^2^ Cu substrate.

The simultaneous formation of the Cu-rGO nanocomposite was carried out in 0.1 M Na_2_SO_4_ via cyclic voltammetry in the potential range between 0.0 to −1.2 V (vs. Ag/AgCl) for five cycles. The prepared Cu-rGO nanocomposites were subsequently rinsed with a copious volume of water and employed for further surface characterization and electrochemical measurements.

For comparison, Cu nanoparticles (NPs) were prepared using the same electrochemical reduction approach in the absence of GO. Briefly, a mixture of CuSO_4_.5H_2_O (10.0 mM) and Nafion (0.5%) in water was prepared and sonicated for 20 minutes. Subsequently, 75 μL of the mixture was cast on the etched Cu foil surface and dried in ambient air at room temperature. The rGO sheet electrode was then prepared by applying the identical conditions mentioned above for the Cu NPs; however, only GO (0.5 mg mL^−1^) with Nafion was used.

### Structural characterization

Morphological surface studies and EDX analysis were carried out using a FE-SEM (Hitachi SU-70). XPS spectra were recorded via a Thermo Fisher XPS system, where the size of the X-ray spot was 400 mm, with an Al Kα monochromatic source. XPSPEAK 4.1 software was used for all of the data processing.

### Electrochemical characterization

Linear Sweep Voltammetry and chronoamperometry were carried out with a CHI660E electrochemical workstation (CH Instrument Inc. USA) utilizing a conventional one-compartment three-electrode cell, whereas a platinum coil (10 cm^2^) was used as the counter electrode. A silver/silver chloride electrode (Ag/AgCl, 3.0 M) was utilized as the reference electrode and all the aforementioned electrode potentials were converted to the reversible hydrogen electrode (RHE) scale using the following equation:

$${\rm{E}}({\rm{v}}{\rm{s}}.\,{\rm{R}}{\rm{H}}{\rm{E}})={\rm{E}}({\rm{v}}{\rm{s}}.\,{\rm{A}}{\rm{g}}/{\rm{A}}{\rm{g}}{\rm{C}}{\rm{l}})+0.210{\rm{V}}+0.0591{\rm{V}}\times {\rm{p}}{\rm{H}}$$

A VoltaLab potentiostat (PGZ-301) was employed for the Electrochemical Impedance Spectroscopic (EIS) measurements, and the frequency was varied from 100 kHz to 10 mHz with an a.c. voltage amplitude of 10 mV. Data acquisition and analyses were performed using Z-view software, which was employed to fit and obtain an equivalent circuit for EIS data. The solution was purged with CO_2_ in order to achieve a CO_2_-saturated condition. All electrochemical experiments were conducted at ambient room temperature (20 ± 2 °C).

### Product analysis

A gas-tight two-compartment electrochemical cell was used for the product formation and analysis. A cationic exchange membrane (CMI-7000S) was utilized as a separator in the cell. Each compartment contained 35.0 ml of the electrolyte; the working electrode and reference electrode (Ag/AgCl) were in the same compartment, whereas the counter electrode was in a separate compartment. Prior to testing, the electrolyte was purged once again with CO_2_ gas for at least 30 minutes. A gas-tight syringe (Hamilton^TM^, 50 μL) was used to transfer the evolved gases into the gas chromatography (Varian 450-GC) to analyze the gas products. The resulting liquid products were qualitatively analyzed using HPLC (Varian Prostar 230 with a Symmetry®C8 column containing dimethyloctylsilyl bonded amorphous silica-acetonitrile). The COD analysis was conducted using 174–334 accu-TEST standard range (5–150 mg/l) twist cap vials for quantitative determination. The solution (2.0 mL) was then transferred to a vial that contained a chromic acid solution, heated to 150 °C for two hours and then allowed to cool. To establish the actual COD values, the results were subtracted from the values of a blank solution (CO_2_ saturated 0.1 M NaHCO_3_ solution). The UV absorbance was recorded at 420 nm using an HACH-DR 2800 portable spectrophotometer.

## Electronic supplementary material

Supplementary information
