# Supplementary material for: Unique copper and reduced graphene oxide nanocomposite toward the efficient electrochemical reduction of carbon dioxide
Source: Sci Rep. 2017 Jun 9;7:3184. doi: 10.1038/s41598-017-03601-3 (PMC5466611; doi:10.1038/s41598-017-03601-3)
Supplement: Supplementary file 1 — Supplementary information [file 41598_2017_3601_MOESM1_ESM.pdf]

# Supplementary information

## Unique copper and reduced graphene oxide nanocomposite toward the efficient electrochemical reduction of carbon dioxide

Md N. Hossain, Jiali Wen, and Aicheng Chen*

Department of Chemistry, Lakehead University, 955 Oliver Road, Thunder Bay, ON P7B 5E1, Canada

**Corresponding Author. E-mail: aicheng.chen@lakeheadu.ca; Fax: +1 807 346 7775; Tel:+1 807 343 8318*

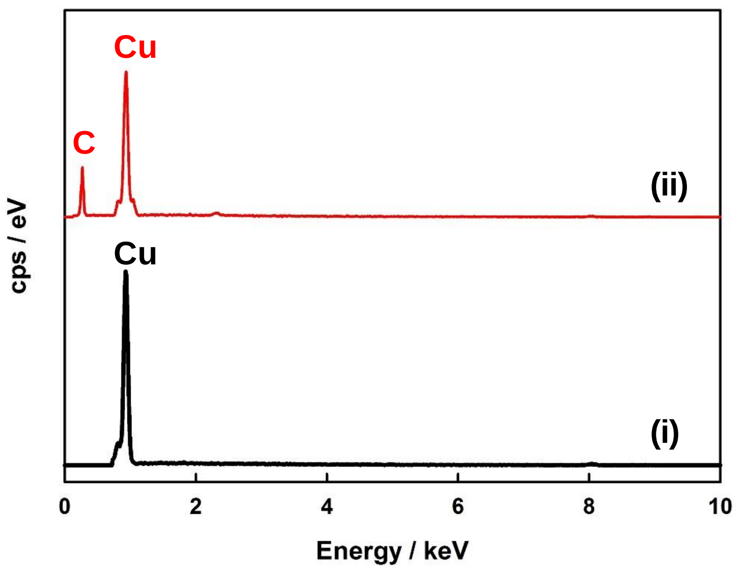


**Supplementary Figure 1.** EDX spectra of the Cu NPs (i) and the Cu-rGO nanocomposite electrode (ii).

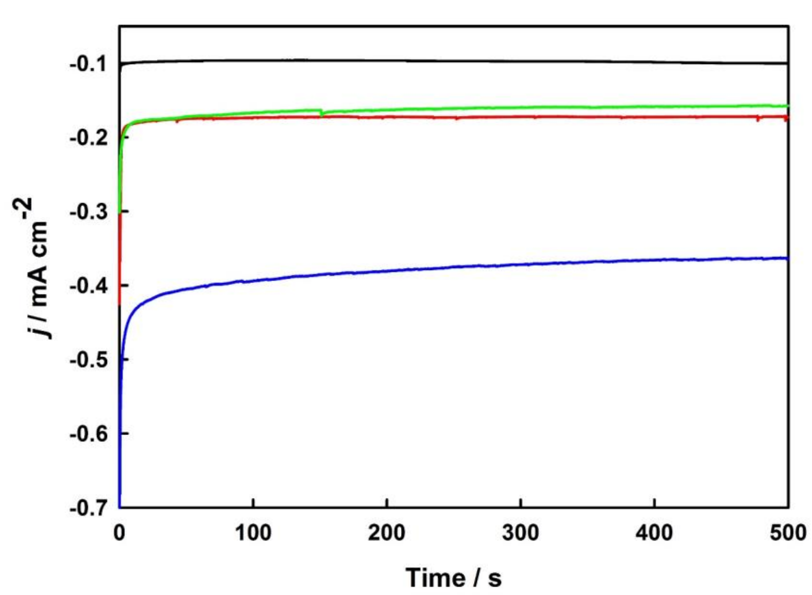


**Supplementary Figure 2.** CA curves of the bare Cu substrate (black), Cu NPs (red), rGO (green), Cu-rGO nanocomposite (blue) electrodes recorded at -0.4 V in a $CO_2$-saturated 0.1 M $NaHCO_3$ solution.

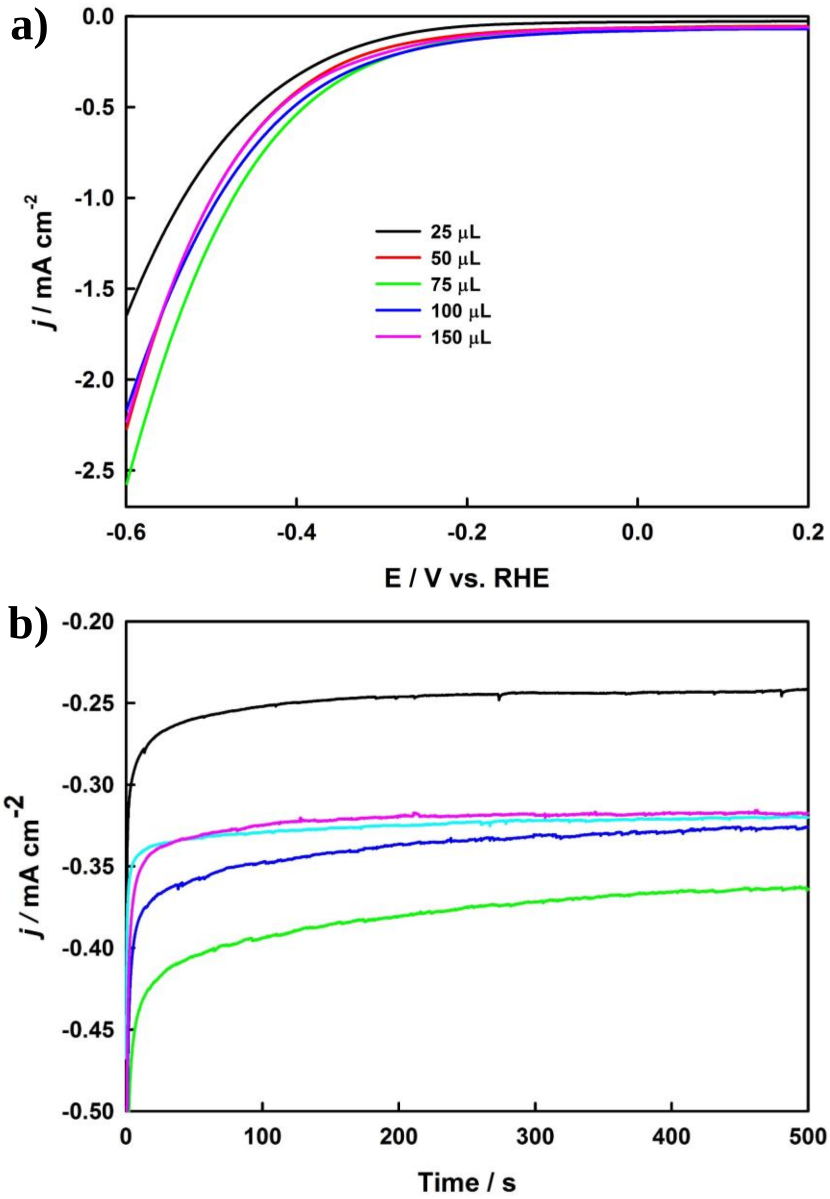


**Supplementary Figure 3.** LSV curves (a) and CA plots (b) of the Cu-rGO nanocomposite electrodes prepared with the optimized GO (0.5 mg $mL^{-1}$) and Cu (10 mM) concentration while the volume of the mixed solution was altered from 25 to 150 μL as listed in Figure S3a.

**Supplementary Table 1.** XPS analysis of C1s peak of the $CuSO_4$-GO thin film before the electrochemical treatment and the formed Cu-rGO nanocomposite.

| XPS | Peak position / eV | Peak assignments | At (%) |
|---|---|---|---|
| Before the electrochemical treatment | 284.80 | $sp^2$ C | 2.17 |
| | 285.76 | C-OH | 26.56 |
| | 286.69 | C-O | 11.70 |
| | 287.95 | C=O | 13.06 |
| | 290.39 | HO-C=O | 46.50 |
| Following the electrochemical treatment | 284.80 | $sp^2$ C | 29.49 |
| | 285.67 | C-OH | 9.02 |
| | 286.46 | C-O | 10.41 |
| | 287.67 | C=O | 11.08 |
| | 290.29 | HO-C=O | 36.99 |

**Supplementary Table 2.** XPS analysis of the $Cu2p^{3/2}$ peak of the $CuSO_4$-GO thin film before the electrochemical treatment and the formed Cu-rGO nanocomposite.

| XPS | Peak position / eV | Peak assignments | At (%) |
|---|---|---|---|
| Before the electrochemical treatment | 934.39 | Cu (II) | 60.52 |
| | 937.08 | | 34.46 |
| | 938.10 | | 5.02 |
| Following the electrochemical treatment | 934.12 | Cu (0) | 59.18 |
| | 931.78 | Cu (I) | 18.77 |
| | 936.82 | Cu (II) | 22.03 |

**Supplementary Table 3.** Values of the elements in equivalent electric circuit fitted in the Nyquist plots of Figure 2b (error percentage for each element is given in parentheses).

| Elements | Bare | rGO | Cu NPs | Cu-rGO |
|---|---|---|---|---|
| Rs ($\Omega$ cm$^{-2}$) | 20.58 (0.46) | 7.82 (0.94) | 15.58 (0.78) | 13.77 (0.67) |
| CPE-T ($\mu$F cm$^{-2}$) | 191.06 (1.60) | 781.40 (3.84) | 427.38 (1.95) | 1817.60 (4.07) |
| CPE-P | 0.90 (0.75) | 0.83 (2.60) | 0.89 (2.93) | 0.80 (3.61) |
| Rct ($\Omega$ cm$^{-2}$) | 668.80 (2.67) | 624.60 (3.27) | 612.90 (2.27) | 355.40 (3.12) |
| W-R ($\Omega$ cm$^{-2}$) | 20.75 (3.89) | 14.75 (2.90) | 18.29 (9.78) | 6.48 (3.02) |
| W-T (s) | 0.007 (4.44) | 0.008 (3.87) | 0.007 (6.90) | 0.013 (3.54) |
| W-P | 0.47 (7.08) | 0.42 (3.33) | 0.42 (1.89) | 0.45 (3.67) |

Rs: solution resistance; CPE-T/CPE-P: elements of constant phase element; Rct: charge transfer resistance; W-R/W-T/W-P: elements of Warburg impedance associated to diffusion resistance.

**Supplementary Table 4.** Values of the elements in equivalent electric circuit fitted in the Nyquist plots of the Supporting Information Figure S4. (Error percentage of each element is given in the parentheses).

| Elements | -0.30 V | -0.4 V | -0.5 V | -0.6 V |
|---|---|---|---|---|
| Rs ($\Omega$ cm$^{-2}$) | 12.88 (0.36) | 13.77 (0.67) | 13.87 (0.45) | 14.03 (0.45) |
| CPE-T ($\mu$F cm$^{-2}$) | 1907 (4.67) | 1817.60 (4.07) | 1785.80 (3.24) | 1700.34 (4.57) |
| CPE-P | 0.86 (2.73) | 0.80 (3.61) | 0.78 (2.61) | 0.76 (2.87) |
| Rct ($\Omega$ cm$^{-2}$) | 976.00 (1.05) | 355.40 (3.12) | 151.00 (3.78) | 70.26 (3.31) |
| W-R ($\Omega$ cm$^{-2}$) | 20.67 (4.56) | 16.48 (3.02) | 14.34 (1.90) | 10.12 (7.44) |
| W-T (s) | 0.008 (2.22) | 0.013 (3.54) | 0.026 (3.42) | 0.043 (2.29) |
| W-P | 0.36 (4.58) | 0.45 (3.67) | 0.45 (4.07) | 0.48 (1.67) |

Rs: solution resistance; CPE-T/CPE-P: elements of constant phase element; Rct: charge transfer resistance; W-R/W-T/W-P: elements of Warburg impedance associated to diffusion resistance.
